# 676. Evaluating Early Mobilization After Split Thickness Skin Grafting and Discharge Disposition in Burn Patients

**DOI:** 10.1093/jbcr/irag033.557

**Published:** 2026-04-06

**Authors:** Maybelle E Singson, Desiree Pinto, Kate F Wallace, Emily G Woolhiser, Katelyn Marzo, Tuan D Le, Bonnie C Carney, Jamie G Oh, Taryn E Travis, Jeffrey W Shupp, Shawn Tejiram, Lauren T Moffatt

**Affiliations:** MedStar Washington Hospital Center, Washington, DC; MedStar Georgetown University Hospital / Washington Hospital Center, Washington, DC; MedStar Washington Hospital Center, Washington, DC; Memorial Hospital System, Davie, FL; Marymount University, Dumfries, VA; The Burn Center at MedStar Washington Hospital Center, Washington, DC; The Burn Center at MedStar Washington Hospital Center, Washington, DC; MedStar Washington Hospital Center, Washington, DC; The Burn Center at MedStar Washington Hospital Center, Washington, DC; MedStar Washington Hospital Center, Washington, DC; MedStar Washington Hospital Center, Washington, DC; The Burn Center at MedStar Washington Hospital Center, Washington, DC

## Abstract

**Introduction:**

Prolonged immobilization as much as fourteen days after split thickness skin grafting (STSG) in burn patients has been described to minimize post-operative complications such as graft loss. Earlier mobilization after STSG is suggested to decrease deconditioning and decrease length of stay. However, there remains a paucity of literature exploring the timing of mobilization following STSG and discharge disposition in burn patients. In this work, it was hypothesized that early mobilization after STSG would be associated with increased incidence of discharge home.

**Methods:**

Adult burn injured patients who presented to an ABA-verified burn center, underwent STSG, and survived to discharge over a three-year period from 2021 to 2024 were retrospectively reviewed. Patients were cohorted by timing of mobilization after their initial incidence of STSG, either early (POD0-1) or late (POD2 or later). Patient electronic medical records were reviewed for demographic information, injury characteristics, and operative timing. Prior level of function, prior living situation, highest level of mobility achieved during the initial therapy session after STSG, and final discharge disposition were collected. Discharge disposition was stratified into two categories: home (home without services, home health, outpatient, or previous living situation) or facility (acute rehab, subacute rehab, and long-term acute care).

**Results:**

During the study period, there were 240 patients who met inclusion criteria. There was no significant difference in demographics, baseline function, prior living situation, or highest level of mobility achieved between mobility groups. Discharge disposition was not significantly different between early and late mobilization groups (*p*=.12). Overall, discharge home was not associated with early vs late mobilization (86.2% vs 81.8%, *p*=.40). A further sub-analysis by age and total body surface area (TBSA) was also performed. When stratified by age, no differences between mobility groups were observed in discharge to home in older patients aged ≥65 years (67.6% vs 46.2%, *p*=.20) or those aged <65 years (91.2% vs 90.6%, *p*>.99). There were also no differences between mobility groups in discharge to home in TBSA burns ≥20% (60.0% vs 50.0%, *p*>.99) or those <20% (89.6% vs 83.9%, *p*=.24). There was no difference in mortality among mobility groups (2.3% vs 1.5%, *p*>.99).

**Conclusions:**

Mobilization timing after STSG had no significant differences in discharge disposition, even when considering age or burn size. Further study should examine functional markers beyond discharge disposition to better understand the impact of rehabilitation timing on outcomes in burn patients.

**Applicability of Research to Practice:**

Burn therapists should utilize a variety of functional outcomes to measure patient response to rehabilitation after STSG.

**Funding for the study:**

N/A.

